# Physiology: A Manual for Students and Practitioners

**Published:** 1900-03

**Authors:** 


Physiology: A Manual for Students and Practitioners. By Howard D. Collins, M. D, Assistant to the Attending Surgeon of the Roosevelt Hospital; Assistant Demonstrator of Anatomy, College of Physicians and Surgeons (Colnmbia University), New York, and Wm. H. Rockwell, Jr., M. D., Assistant Demonstrator of Anatomy, College of Physicians and Surgeons (Columbia University), N. Y.; Member of Association of American Anatomists. Series edited by Bern B. Gallaudet, M. D., Demonstrator of Anatomy and Instructor in Surgery, College of Physicians and Surgeons, Columbia University, New York; Visiting Surgeon, Bellevue Hospital, New York. Illustrated wiih 153 engravings. 12mo, pp. 323. Philadelphia and New York: Lea Brothers & Co. 1899.

This volume belongs in Lea's series of pocket text-books, mention of which has been made previously in the Journal. It holds a place intermediate between the quiz-compend and the treatise on physiology. It is well printed and illustrated and gives as full an account of the subjects covered in the term physiology, as is to be expected in a work of its size and character. The style of the authors has the merit of clearness, and the arrangement of matter is good. The medical student will undoubtedly find it useful and superintendents of hospital training schools, when looking for a book to put into the hands of pupi! nurses will do well to examine this little volume. M. J. F.

